# PKM2 promotes angiotensin‐II‐induced cardiac remodelling by activating TGF‐β/Smad2/3 and Jak2/Stat3 pathways through oxidative stress

**DOI:** 10.1111/jcmm.17007

**Published:** 2021-10-23

**Authors:** Xiyu Zhang, Cuiting Zheng, Zhenqiang Gao, Lingling Wang, Chen Chen, Yuanyuan Zheng, Yan Meng

**Affiliations:** ^1^ Beijing Key Laboratory of Metabolic Disorders Related Cardiovascular Diseases Beijing Lab for Cardiovascular Precision Medicine Department of Pathology Capital Medical University Beijing China; ^2^ Department of Pathology Beijing Shijitan Hospital Capital Medical University Beijing China; ^3^ China‐America Institute of Neuroscience Beijing Luhe Hospital Capital Medical University Beijing China; ^4^ Department of Pharmacology Capital Medical University Beijing China

**Keywords:** Ang II, cardiac remodelling, oxidative stress, PKM2, shikonin

## Abstract

Hypertensive cardiac remodelling is a common cause of heart failure. However, the molecular mechanisms regulating cardiac remodelling remain unclear. Pyruvate kinase isozyme type M2 (PKM2) is a key regulator of the processes of glycolysis and oxidative phosphorylation, but the roles in cardiac remodelling remain unknown. In the present study, we found that PKM2 was enhanced in angiotensin II (Ang II)‐treated cardiac fibroblasts and hypertensive mouse hearts. Suppression of PKM2 by shikonin alleviated cardiomyocyte hypertrophy and fibrosis in Ang‐II‐induced cardiac remodelling in vivo. Furthermore, inhibition of PKM2 markedly attenuated the function of cardiac fibroblasts including proliferation, migration and collagen synthesis in vitro. Mechanistically, suppression of PKM2 inhibited cardiac remodelling by suppressing TGF‐β/Smad2/3, Jak2/Stat3 signalling pathways and oxidative stress. Together, this study suggests that PKM2 is an aggravator in Ang‐II‐mediated cardiac remodelling. The negative modulation of PKM2 may provide a promising therapeutic approach for hypertensive cardiac remodelling.

## 1 INTRODUCTION

Hypertension is a key risk cause for cardiovascular diseases, remaining the predominant cause of mortality in the world and presenting a considerable economic burden.^1^ Pressure overload induces cardiac remodelling that includes cardiomyocyte hypertrophy and fibrosis, and ultimately leads to the progression of heart failure.^2^ As the predominant effector of the renin‐angiotensin‐aldosterone system (RAAS), angiotensin II (Ang II) promoted cardiac fibrosis by inducing fibroblasts proliferation and stimulating collagen synthesis and eventually caused cardiac remodelling and heart failure.^3^

Multiple signalling pathways have been demonstrated to positively regulate cardiac fibrosis, including transforming growth factor β (TGF‐β)/small mothers against decapentaplegic proteins 2 and 3 (Smad2/3), Janus kinase 2 (Jak2)/signal transducer and activator of transcription 3 (Stat3) signalling pathways.^4^ TGF‐β induced phosphorylation of Smad2 and Smad3 to promote extracellular matrix secretion which plays important roles in myocardial fibrosis.^5^ Moreover, activation of Jak2/Stat3 signalling pathway enhances collagen‐related gene transcription to accelerate fibrosis.^6^ Hence, targeting these signalling pathways might provide new therapeutic strategies to reverse cardiac remodelling.

Oxidative stress, defined as disturbances in the pro‐/anti‐oxidant balance, is harmful to cells due to an excess production of reactive oxygen species (ROS).^7^ Accumulating evidence indicates that oxidative stress plays a vital role in renal fibrosis, cardiovascular disease and other diseases.^8^, ^9^, ^10^ It is reported that ROS mediates Ang‐II‐induced fibrotic cardiomyopathy by activating transcription factors and fibrotic signalling kinases.^11^ Besides, glutathione (GSH) and GSH peroxidase 4 (GPx4) act as negative regulators of oxidative stress by limiting ROS production.^12^

Pyruvate kinase muscle isozyme 2 (PKM2) is an isoenzyme of the glycolytic enzyme pyruvate kinase. The primary function of PKM2 is to mediate the conversion of phosphoenolpyruvate (PEP) to pyruvate as the last step of glycolysis to generate ATP.^13^ PKM2 also has various biological functions, including proinflammatory cytokine production, cells proliferation and oxidative stress.^14^, ^15^, ^16^ However, the potential role of PKM2 in Ang‐II‐induced cardiac remodelling remains unknown.

To explore the potential role of PKM2 in cardiac remodelling diseases, we analysed the expression of PKM2 in Ang‐II‐treated neonatal rat cardiac fibroblasts (NRCFs), neonatal rat cardiomyocytes (NRCMs) and mouse hearts. Furthermore, we checked the role of PKM2 in cardiac remodelling. Finally, we elucidated the potential underlying mechanism of PKM2 in the development of cardiac remodelling.

## 2 METHODS

### 2.1 Antibody and reagents

Antibodies against PKM2 (4053, 1:1000), Jak2 (3230, 1:1000), phosphor‐Jak2 (8082, 1:500), Stat3 (9132, 1:1000), phosphor‐Stat3 (9145, 1:1000), TGF‐β (3711, 1:1000), Smad2/3 (8685, 1:1000), phosphor‐Smad2/3 (8828, 1:1000), S6 (2217s, 1:1000), phosphor‐S6 (2211s, 1:5000) and GAPDH (2118,1:10000) were from Cell Signaling Technology (Danvers, MA, USA). Rapamycin was from MedChem Express (HY‐10219, New Jersey, USA). Shikonin was from Selleck (S8279, Houston, TX, USA). N‐acetyl‐L‐cysteine (NAC) was from Beyotime Institute of Biotechnology (S0077, Shanghai, China).

### 2.2 Animal model

Male C57BL/6 mice were purchased from the Vital River Laboratory Animal Technology Company of Beijing in China. All animal experiments were approved by the Institutional Animal Care and Use Committee of Capital Medical University. Hypertensive cardiac remodelling was induced in 8‐ to 12‐week‐old male mice by chronic subcutaneous infusion of Ang II (A9525, Sigma‐Aldrich) at a dose of 1,500 ng/kg/min using the ALZET^®^ Osmotic Pumps (Model 1007D) for 7 days or 1,000 ng/kg/min using the pumps (Model 1002D) for 14 days. The control mice were infused with saline for 7 or 14 days. For shikonin treatment, mice were randomized to be intraperitoneally injected with either vehicle or shikonin at a dose of 1.25 mg/kg 3 times per week. All mice were analysed by echocardiography and haemodynamic measurements at 7 days or 14 days.

### 2.3 Primary culture of NRCMs and NRCFs

Cardiomyocytes and fibroblasts were isolated from 1‐day‐old SD rat hearts using a conventional method as described.^17^ Briefly, heart tissues were digested by 0.1% trypsin and 0.05% type II collagenase. The dissociated cells in DMEM/F‐12 medium with 15% FBS and 1% penicillin and streptomycin were plated to obtain adhered cardiac fibroblasts after 90 min in incubator (5% CO_2_), and the cardiomyocytes in suspension were transferred to another dish with coating laminin. Both cardiomyocytes and fibroblasts were cultured in serum‐free DMEM/F‐12 for 12 h before experiments.

### 2.4 Transfection

SiRNA was used to knock down the PKM2 in NRCFs. The siRNAs were synthesized by HanBio Biotechnology (Shanghai, China). The sequence of PKM2 siRNA was: 5′‐GCAAGAACAUCAAGAUCAU‐3′. The sequence of negative control siRNA was as follows: 5′‐UUCUCCGAACGUGUCACGU‐3′. Transfections of the siRNAs were performed by using Lipofectamine RNAiMAX Reagent (Thermo Fisher Scientific) according to the manufacturer's protocol. Briefly, cells were seeded in 6‐well plates to obtain approximately 30%–50% confluence. Twenty microlitres of mixture containing siRNA (20 pM for each) and transfection reagents (5 μl for each) were added to the cells for 6 h at 37℃ and then replaced with fresh culture medium. The efficiency of transfection was confirmed by Western blot analysis.

### 2.5 Cell migration assay

Cell migration was examined by wound healing assay. NRCFs grown to 90% in 6‐well plates were scratched via a 200 μl pipette tip to create the wound followed by washing detached cells with PBS. Cultured cells were grown for 12 h and 24 h to allow wound healing. Wound healing area was measured using Image J (National Institutes of Health, Bethesda, MD, USA), and % of wound area was analysed by GraphPad Prism 7.0 (GraphPad Software). % of wound area represents the ration of wound area at T_12h_ (or T_24h_)/wound area at T_0h_.

### 2.6 Cell counting assay

The cell viability was detected by cell counting kit‐8 (CCK‐8) (YEASEN, Shanghai, China). Briefly, 10 μl of CCK‐8 solution was added to each well and incubated at 37℃ for 2.5 h; then, the OD value for each well was read at wavelength 450 nm with a microplate reader (Multiskan, Thermo, USA). The assay was repeated 3 times. The cell viability was calculated as follows:

$$cell\;viability\left(\%\right)=\frac{\text{OD}\left(\text{experiment}\right)‐\text{OD}\left(\text{blank}\right)}{\text{OD}\left(\text{control}\right)‐\text{OD}\left(\text{blank}\right)}\times 100$$

### 2.7 Cell oxidative stress assays

Reactive oxygen species levels were determined by Flow cytometry. Briefly, 5 × 10^5^ NRCFs with pretreatment of 0.1 μM shikonin or DMSO for 2 h were treated with Ang II for 12 h, and then incubated with 10 μM DCFH‐DA for 20 min at 37℃. After washing 3 times with PBS, cells were harvested and analysed for the fluorescence of DCFH by flow cytometry (BD Accuri C6, Frankin Lakes, NJ, USA). MDA levels in cells were measured using the MDA assay kit (Beyotime) according to the manufacturer's instructions, and MDA concentration was calculated in μmol per gram of protein. Total glutathione (GSH+GSSG) and GSSG were measured according to the GSH and GSSG assay kit (Beyotime) using kinetic determination methods under a 412 nm wavelength, and the GSH/GSSG ratio was calculated.

### 2.8 Pyruvate production assay

Pyruvate production was detected using PA assay kit (Solarbio, Beijing, China). Briefly, 5 × 10^5^ NRCFs were treated with Ang II and then washed 3 times with PBS and were harvested with 1 ml extracting solution. After ultrasonic disruption, cells were freezing for 30 min and then centrifuged to collect the supernatant. Pyruvate levels in mouse serums and NRCFs were measured using the kit according to the manufacturer's instructions.

### 2.9 Measurement of blood pressure

Mice were acclimatized for 7 days prior to experimentation. The blood pressure of all mice was measured before Ang II infusion and every day in 7‐day model or every other day in 14‐day model. Systolic blood pressure (SBP) was recorded when were mice under a conscious condition by tail‐cuff method with the CODA noninvasive BP system (Kent Scientific Corporation).

### 2.10 Echocardiography

Mice were lightly anaesthetized with 1.5% isoflurane. Cardiac function was evaluated by the high‐resolution Micro‐Ultrasound system (Vevo 770, VisualSonics, Toronto, Ontario, Canada). Briefly, cardiac contractile function and structure were evaluated by M‐mode echocardiography. Ventricular parameters including diastolic left ventricular anterior wall (LVAW;d) and systolic left ventricular anterior wall (LVAW;s) were measured. All the measurements were made from more than three beats and averaged. The ejection fraction (EF %) and fractional shortening (FS %) were calculated to judge the cardiac systolic function.

### 2.11 Histopathology analysis

For histological analysis, hearts were fixed, embedded and sectioned into 5‐μm‐thick slices. Sections were used to stain wheat germ agglutinin (WGA) to assess cellular hypertrophy. Sections were stained with Masson's trichrome (collagen, blue; cytoplasm, red/pink) for collagen deposition analysis. Immunohistochemistry images were captured with Pannoramic SCAN II (3DHISTECH Ltd) and analysed by using Image J.

### 2.12 Quantitative RT‐PCR (qRT‐PCR) analysis

Total RNA was extracted from the heart tissues and cells by using Trizol method (T9424, Sigma‐Aldrich). The first‐strand cDNA was synthesized from 1 μg of total RNA by HIScript‐II Q RT SuperMix for Qpcr (Vazyme). qRT‐PCR was performed using SYBR Green Master Mix (Transgen, Beijing, China) with an iCycler IQ system (Bio‐Rad Laboratories, Inc Hercules, CA, USA). qRT‐PCR was cycled in 94℃/5 s and 60℃/60 s for 40 cycles, after an initial denaturation step at 94℃ for 30 s. The expression of mRNA was normalized to the amount of endogenous control (GAPDH). All samples were run in duplicate. The following primers for genes:

#### 2.12.1 Mouse:

GAPDH: 5′‐GGTTGTCTCCTGCGACTTCA‐3′; 5′‐GGTGGTCCAGGGTTTCTTACTC‐3′;

ANF: 5′‐CACAGATCTGATGGATTTCAAGA‐3′; 5′‐CCTCATCTTCTACCGGCATC‐3′;

BNP: 5′‐GAAGGTGCTGTCCCAGATGA‐3′; 5′‐CCAGCAGCTGCATCTTGAAT‐3′;

Collagen I: 5′‐GAGTACTGGATCGACCCTAACCA‐3′; 5′‐ACGGCTGAGTAGGGAACACA‐3′;

Collagen III: 5′‐TCCCCTGGAATCTGTGAATC‐3′; 5′‐TGAGTCGAATTGGGGAGAAT‐3′;

PKM2: 5′‐GTCTGGAGAAACAGCCAAGG‐3′; 5′‐CGGAGTTCCTCGAATAGCTG‐3′;

GPx4: 5′‐TGTGGTTTACGAATCCTGGC‐3′; 5′‐CCCTTGGGCTGGACTTTCAT‐3′;

SOD2: 5′‐GCCTGCACTGAAGTTCAATG‐3′; 5′‐ATCTGTAAGCGACCTTGCTC‐3′;

#### 2.12.2 Rat:

GAPDH: 5′‐CCCCCAATGTATCCGTTGTG‐3′; 5′‐TAGCCCAGGATGCCCTTTAGT‐3′;

Collagen I: 5′‐TGTCGATGGCTGCTCCAAAA‐3′; 5′‐AGGCGAGATGGCTTATTCGT‐3′;

Collagen III: 5′‐AGTGGCCATAATGGGGAACG‐3′; 5′‐CAGGGTTTCCATCCCTTCCG‐3′;

PKM2: 5′‐TCTACGTGGACGATGGGCT‐3′; 5′‐AGGAAGACCTTCTCTGCCGGA‐3′;

GPx4: 5′‐GCAACCAGTTCGGGAGGCAGGAG‐3′; 5′‐CCTCCATGGGACCATAGCGCTTC‐3′.

### 2.13 Western blot analysis

Cells or heart tissues were lysed with RIPA lysis buffer. Equal amounts of protein were loaded, separated by SDS‐PAGE gels and transferred to a membrane (Millipore). The membrane was blocked and incubated with primary antibody at 4℃ overnight, and then incubated with secondary antibodies for 1 h at room temperature. Western blot signals were detected with the enhanced chemiluminescence system (Millipore), and signal intensities were analysed with Image J.

### 2.14 Statistical analysis

All data were expressed as mean ± standard error of the mean (SEM) and were analysed using GraphPad Prism 7.0 (GraphPad Software) or SPSS Version 21 software. Comparison between two groups was conducted using two‐tailed, unpaired Student's *t*‐test. In the result with more than two groups, analysis of variance (ANOVA) was applied to analyse the difference. Two‐way ANOVA moderated by Bonferroni post hoc test was used to detect variation between 3 or more groups. Statistical significance was defined as *p *< 0.05.

## 3 RESULTS

### 3.1 PKM2 expression is upregulated in hypertensive heart

**FIGURE 1:**
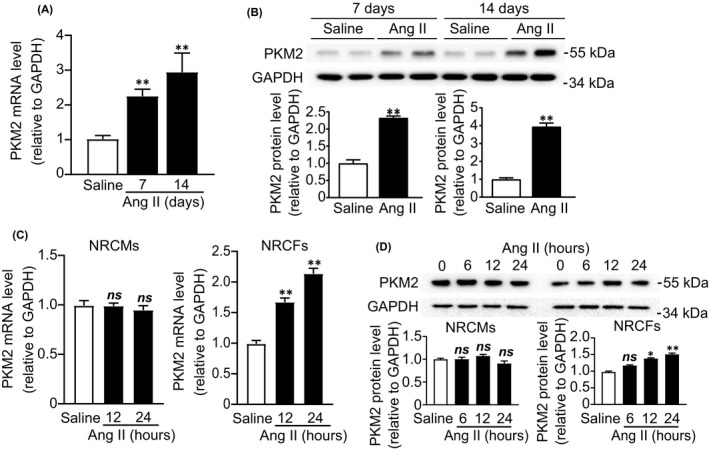
PKM2 expression is upregulated in hypertensive heart. Mice were infused with Ang II (1,000 ng/kg/min) for 7 and 14 days (*n* = 5 per group) (A, B). (A) qRT‐PCR analysis of PKM2 mRNA. (B) Western blot and quantification of PKM2. NRCMs and NRCFs were treated with Ang II (1 μM) for various times (*n* = 3 per group) (C, D). (C) qRT‐PCR analysis of PKM2 mRNA. (D) Western blot and quantification of PKM2. ^*^p < 0.05,^**^p < 0.01

To explore the role of PKM2 in cardiac remodelling, we examined the expression of PKM2 in Ang‐II‐induced hypertensive mouse hearts. PKM2 was boosted at both mRNA and protein levels after 7‐ and 14‐day Ang II infusion (Figure 1A, B). In vitro, PKM2 upregulated in NRCFs in a time‐dependent manner after Ang II treatment (Figure 1C). However, PKM2 expression was not altered in Ang‐II‐treated NRCMs (Figure 1D). Consequently, accumulation of PKM2 led to increase pyruvate production in Ang‐II‐treated hypertensive mouse serums and NRCFs (Figure S1A, B). In summary, these results suggest that PKM2 is boosted in response to fibrotic stimuli and may play a role in Ang‐II‐induced myocardial fibrosis. It is reported that mTOR as a key activator of PKM2 is known augmented by mTOR activation and reduced by mTOR suppression in cancer cells.^18^ To determine whether mTOR involved in the regulation of PKM2, we used rapamycin, inhibitor of mTOR signalling pathway, in Ang‐II‐incubated NRCFs. We found that upregulation of PKM2 induced by Ang II was reduced by rapamycin. (Figure S1C). These data indicate that PKM2 is positively regulated by mTOR.

### 3.2 Inhibition of PKM2 alleviates Ang‐II‐induced early cardiac remodelling

**FIGURE 2:**
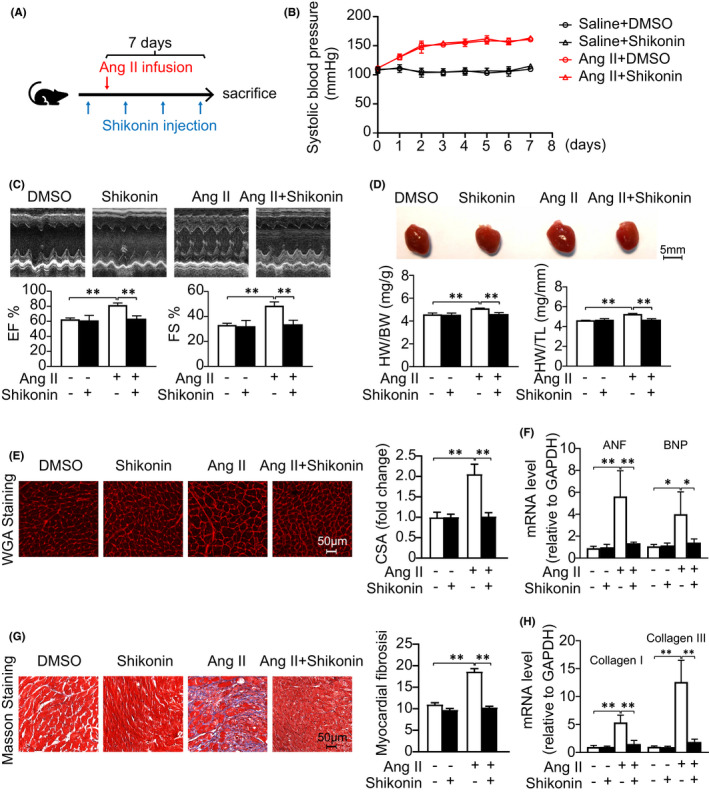
Inhibition of PKM2 alleviates Ang‐II‐induced early cardiac remodelling. DMSO or shikonin was given to C57BL/6J mice while the treatment was combined with Ang II (1,500 ng/kg/min) infusion for 7 days. (A) Diagrammatic illustration of experimental schemes and timelines. (B) Systolic blood pressures. (C) Representative M‐mode echocardiography of the left ventricle (left). Measurement of ejection fraction (EF %) and fractional shortening (FS %) (right, *n *= 6 per group). (D) Representative hearts (left). HW/BW and HW/TL ratios (right, *n* = 6 per group). (E) TRITC‐labelled wheat germ agglutinin (WGA) staining of heart sections. Scale bar, 50 μm (left). Quantification of the relative myocyte cross‐sectional area (right, *n* = 6 per group). (G) Masson's staining of cardiac sections. Scale bar, 50 μm (left). Quantification of the relative fibrosis (right, *n* = 6 per group). (F, H) qRT‐PCR analysis of mRNA expression (*n* = 6 per group). ^*^p < 0.05, ^**^p < 0.01

To determine whether enhanced PKM2 causes adverse cardiac remodelling in early‐stage, wild‐type (WT) mice were treated with PKM2 inhibitor, shikonin (1.25 mg/kg, i.p., 3 times per week) and Ang II (1,500 ng/min/kg) for 7 days (Figure 2A). PKM2 was downregulated after treatment of shikonin (Figure S2A). Systolic blood pressure induced by Ang II infusion was not influenced by shikonin (Figure 2B). In Ang‐II‐induced 7‐day mouse cardiac remodelling, shikonin recovered cardiac pump function, which included left ventricular ejection fraction (EF %) and fractional shortening (FS %) (Figure 2C). Concomitantly, shikonin decreased Ang‐II‐induced hypertrophic responses, including heart weight/body weight (HW/BW) ratio, heart weight/tibia length (HW/TL) ratio, the cross‐sectional area of myocytes and the expression of hypertrophic markers atrial natriuretic factor (ANF) and brain natriuretic peptide (BNP) (Figure 2D–F). In addition, shikonin relieved Ang‐II‐induced an increase in the area of cardiac fibrosis and collagen I and III expression (Figure 2G, H). Hence, inhibition of PKM2 significantly inhibits Ang‐II‐induced early cardiac remodelling and rescues cardiac function.

### 3.3 PKM2 inhibition ameliorates Ang‐II‐induced late cardiac remodelling

**FIGURE 3:**
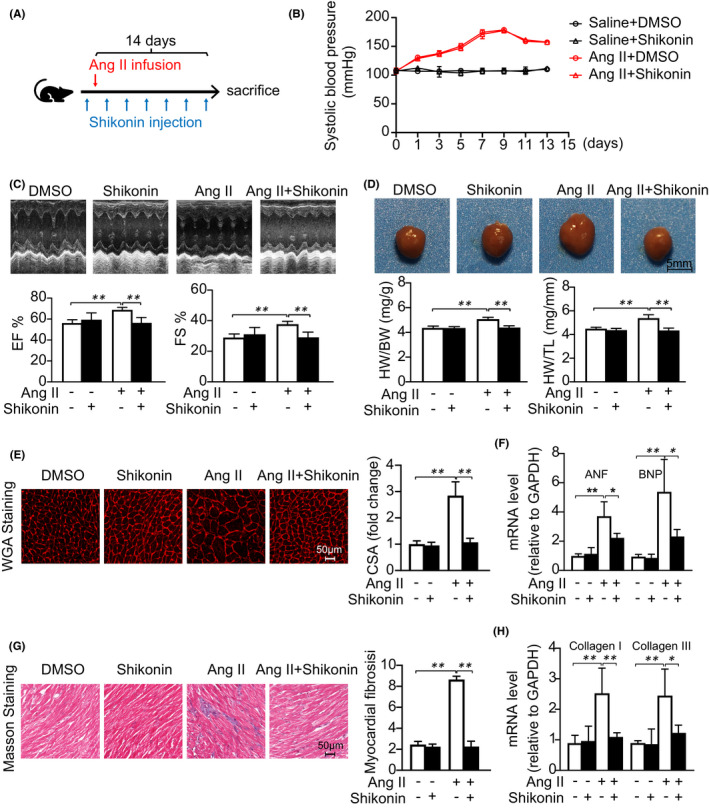
PKM2 inhibition ameliorates Ang‐II‐induced late cardiac remodelling. DMSO or shikonin was given to C57BL/6J mice 3 times per week while the treatment was combined with Ang II (1,000 ng/kg/min) infusion for 14 days. (A) Diagrammatic illustration of experimental schemes and timelines. (B) Systolic blood pressures. (C) Representative M‐mode echocardiography of the left ventricle (left). Measurement of ejection fraction (EF %) and fractional shortening (FS %) (right, *n* = 6 per group). (D) Representative hearts (left). HW/BW and HW/TL ratios (right, *n* = 6 per group). (E) TRITC‐labelled wheat germ agglutinin (WGA) staining of heart sections. Scale bar, 50 μm (left). Quantification of the relative myocyte cross‐sectional area (right, *n* = 6 per group). (G) Masson's staining of cardiac sections. Scale bar, 50 μm (left). Quantification of the relative fibrosis (right, *n* = 6 per group). (F, H) qRT‐PCR analysis of mRNA expression (*n* = 6 per group). ^*^p < 0.05, ^**^p < 0.01

Furthermore, we treated WT mice with shikonin and Ang II (1,000 ng/min/kg) for 14 days in late cardiac remodelling model (Figure 3A). Consistently with 7‐day model, PKM2 was downregulated after treatment of shikonin (Figure S2B). Accordingly, systolic blood pressure increased by Ang II infusion was not altered by shikonin (Figure 3B), and shikonin reduced cardiac hypertrophy/fibrosis and improved cardiac function with Ang II infusion (Figure 3C–H). Consistently, inhibition of PKM2 can also reverse late cardiac dysfunction and remodelling in Ang‐II‐infused mice.

### 3.4 Inhibition of PKM2 suppresses Ang‐II‐induced cardiac fibroblasts dysfunction

**FIGURE 4:**
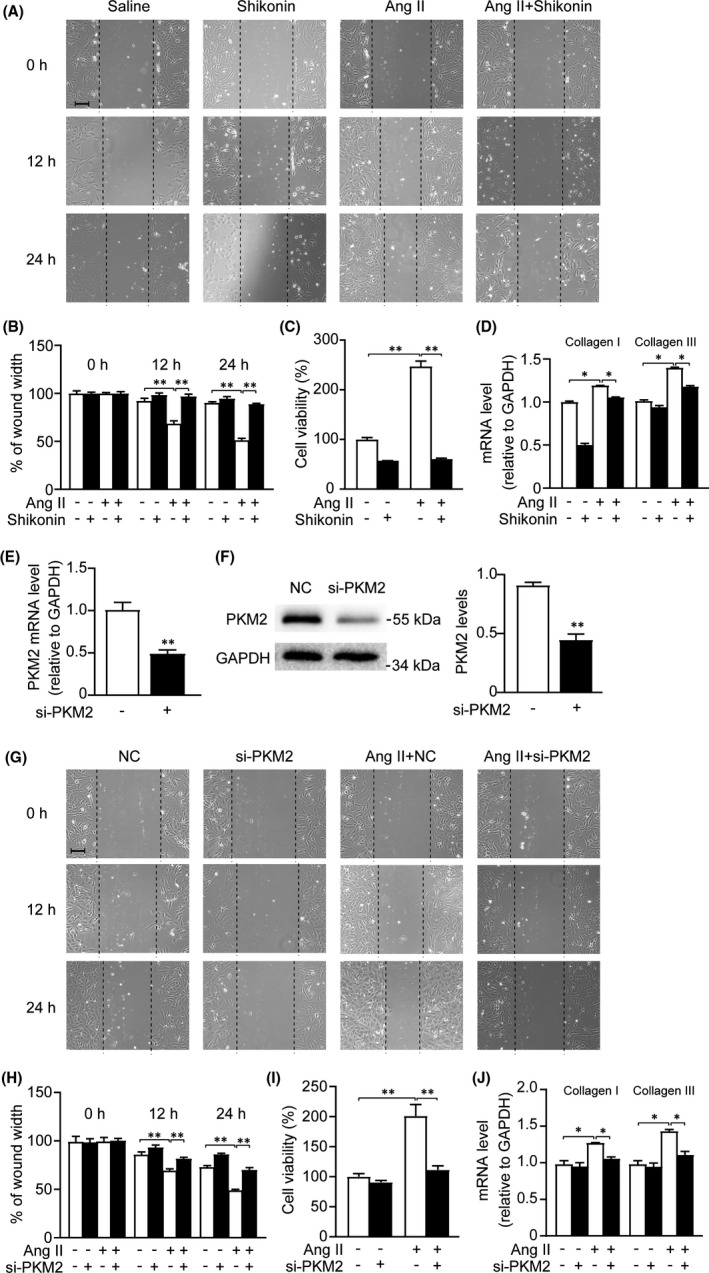
Inhibition of PKM2 suppresses Ang‐II‐induced cardiac fibroblasts dysfunction. NRCFs were treated with shikonin (0.1 μM) and with/out Ang II (1 μM) for 24 h (for A, B and C) or 48 h (for D). And NRCFs were transfected with negative control or si‐PKM2 (for E–J) and with/out Ang II for 24 h (for G, H and I) or 48 h (for J). (A and G) Scratch wound healing assay. (B and H) Cell migration rate was quantified by measuring the difference in area between the leading edge at the initiation of the experiment at the indicated times. Scale bar, 50 μm. (*n* = 3 per group). (C and I) CCK‐8 assay was used to examine the cell viability (*n* = 6 per group). (D and J) qRT‐PCR analysis of mRNA expression (*n* = 3 per group). (E) qRT‐PCR analyses of PKM2 expression (*n* = 3 per group). (F) Western blot and quantification of PKM2 expression. ^*^p < 0.05, ^**^p < 0.01

We next determined the effect of PKM2 inhibition on cardiac fibroblasts migration, proliferation and collagen synthesis in vitro. Scratch wound healing assay showed that inhibition of PKM2 by shikonin significantly attenuated Ang‐II‐induced NRCFs migration (Figure 4A, B). In addition, Ang‐II‐induced NRCFs proliferation and collagen synthesis were dramatically reduced by shikonin (Figure 4C, D). To further clarify the role of PKM2 in cardiac fibrosis, we synthesized the siRNAs to knockdown PKM2 in NRCFs (Figure 4E, F). Consistently, si‐PKM2 also reversed Ang‐II‐induced detrimental effects in NRCFs (Figure 4G–J). Therefore, we suggest PKM2 as a pro‐fibrotic role in Ang‐II‐induced cardiac fibroblasts dysfunction.

### 3.5 **PKM2** **suppression inhibits activation of TGF‐β/Smad2/3 and Jak2/Stat3 signalling pathways**

**FIGURE 5:**
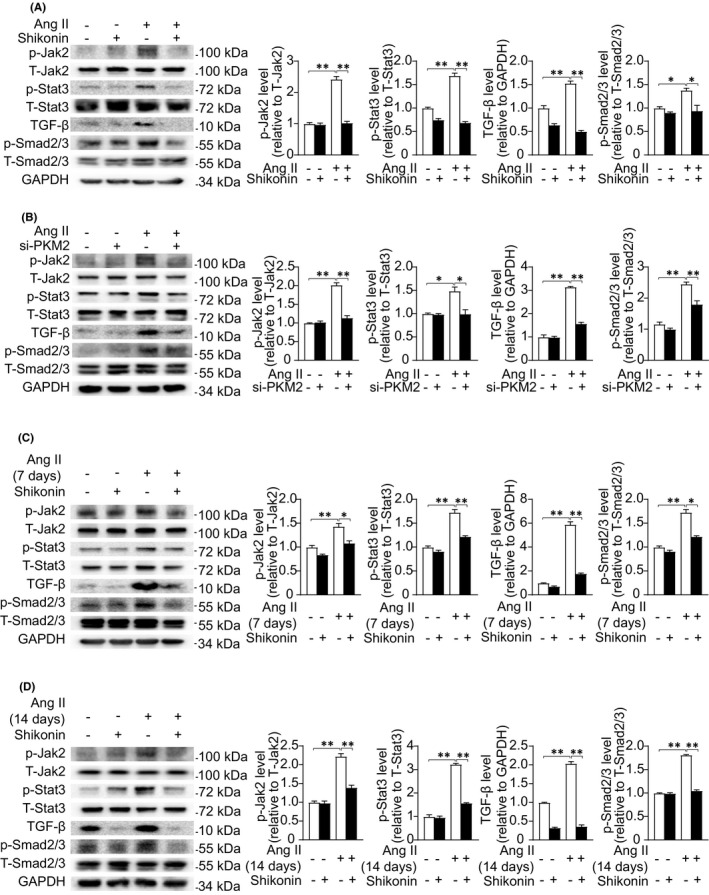
PKM2 suppression inhibits activation of TGF‐β/Smad2/3 and Jak2/Stat3 signalling pathways. (A) Representative Western blotting (left) and quantification (right) showing the expression of the indicated signalling pathways in NRCFs treated with DMSO or shikonin (0.1 μM) for 2 h and with/out Ang II (1 μM) for 5 min. (B) Representative Western blotting (left) and quantification (right) showing the expression of the indicated signalling pathways in NRCFs transfected with negative control or si‐PKM2 for 24 h and with/out Ang II (1 μM) for 5 min. (C) Representative Western blotting (left) and quantification (right) showing the expression of the indicated signalling pathways in mouse heart treated as in Figure 2. (D) Representative Western blotting (left) and quantification (right) showing the expression of the indicated signalling pathways in mouse heart treated as in Figure 3

TGF‐β/Smad2/3 and Jak2/Stat3 are classical signalling pathways involved in cardiac fibrosis. Ang‐II significantly induced increase of TGF‐β and phosphorylation of Smad2/3, Jak2 and Stat3 in cardiac fibroblasts and tissues. To illustrate the underlying molecular mechanisms of the pro‐fibrotic effect of PKM2, we examined whether TGF‐β/Smad2/3 and Jak2/Stat3 signalling pathways were involved in these processes. We found that inhibition of PKM2 by shikonin dramatically decreased the activation of TGF‐β/Smad2/3 and Jak2/Stat3 induced by Ang II (Figure 5A). Consistently, suppression of PKM2 by siRNA also inhibited the activation of TGF‐β/Smad2/3 and Jak2/Stat3 pathways (Figure 5B). Furthermore, shikonin reversed Ang‐II‐induced TGF‐β/Smad2/3 and Jak2/Stat3 activation both in 7‐ and 14‐day remodelling mouse hearts (Figure 5C, D). According to these results, we speculate that PKM2 regulates Ang‐II‐induced pathological cardiac fibrosis through activating TGF‐β/Smad2/3 and Jak2/Stat3 signalling pathways.

### 3.6 Inhibition of PKM2 improves Ang‐II‐induced oxidative stress

**FIGURE 6:**
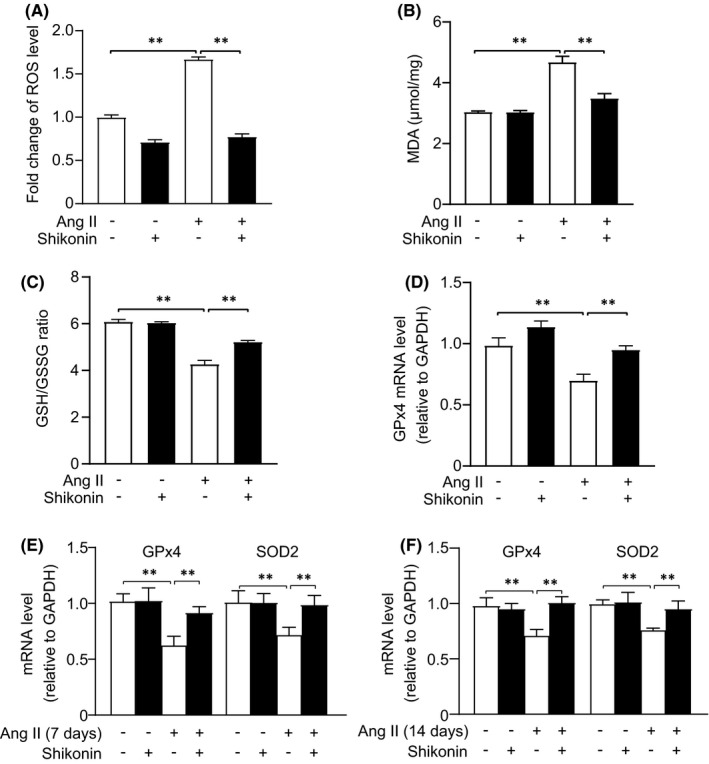
Inhibition of PKM2 improves Ang‐II‐induced cardiac fibroblasts oxidative stress. (A–D) After the treatment of shikonin and Ang II for 12 h in cardiac fibroblasts, ROS, MDA and GSH/GSSG ratio were determined using assay kits, and Gpx4 levels were measured by qRT‐PCR assay (*n* = 3 per group)

To further demonstrate the molecular mechanisms of pro‐fibrotic effect of PKM2, we checked oxidative stress in these processes. DCFH‐DA ROS intensity demonstrated that shikonin dramatically antagonized Ang‐II‐induced oxidative stress in NRCFs (Figure 6A). MDA, the product of oxidative stress showed a similar change to generation of ROS (Figure 6B). We also evaluated anti‐oxidants GSH/GSSG ratio and GPx4. As shown in Figure 6C, D, shikonin treatment significantly increases Ang‐II‐induced GSH/GSSG ratio and GPx4 in NRCFs downregulation. Furthermore, we detected oxidative stress in Ang‐II‐treated 7‐ and 14‐day mouse hearts. Our data showed shikonin treatment significantly improved Ang‐II‐induced GPx4 and SOD2 downregulation (Figure 6E, F). Hence, suppression of PKM2 significantly alleviates Ang‐II‐induced oxidative stress in cardiac remodelling.

### 3.7 PKM2 regulates TGF‐β/Smad2/3 and Jak2/Stat3 signalling pathways through oxidative stress

**FIGURE 7:**
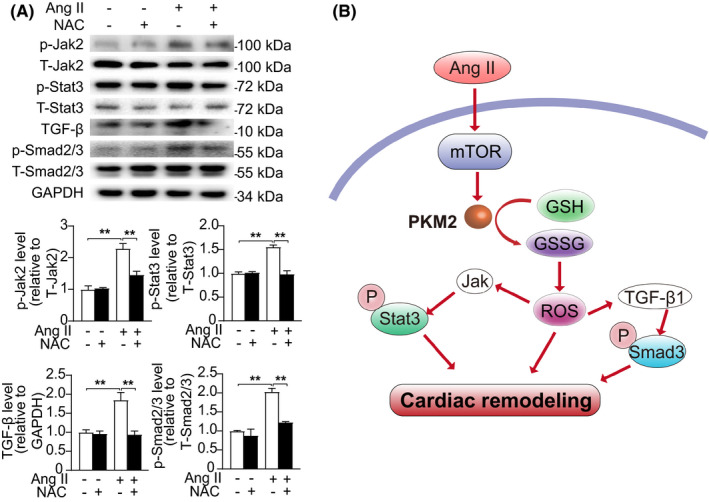
PKM2 regulates TGF‐β/Smad2/3 and Jak2/Stat3 signalling pathways through oxidative stress. (A) Representative Western blotting (up) and quantification (down) showing the expression of the indicated signalling pathways in NRCFs treated with water or NAC (10 μM) for 2 h and with/out Ang II (1 μM) for 5 min. (B) Diagrammatic illustration of PKM2 in cardiac remodelling

It is known that Ang‐II‐induced oxidative stress promotes the activation of TGF‐β/Smad2/3 signalling pathways.^19^, ^20^ Activation of Jak2/Stat3 also required oxidative stress.^21^, ^22^ To explore the potential crosstalk between PKM2 and TGFβ/Smad2/3 and Jak2/Stat3, we used N‐acetylcysteine (NAC), ROS inhibitor targeting oxidative stress. In present study, we found that inhibition of oxidative stress by NAC suppresses Ang‐II‐induced TGFβ/Smad2/3 and Jak2/Stat3 activation in NRCFs (Figure 7A). These data indicate that PKM2 regulates Ang‐II‐induced cardiac fibrosis via TGF‐β/Smad2/3 and Jak2/Stat3 signalling pathways through oxidative stress.

## 4 DISCUSSION

In this study, we identified PKM2 as a detrimental factor in pathological cardiac remodelling. PKM2 was increased in both Ang‐II‐treated hearts and NRCFs, but not in NRCMs. Inhibition of PKM2 in mice alleviated Ang‐II‐induced cardiac remodelling and dysfunction. In vitro, suppression of PKM2 in NRCFs attenuated Ang‐II‐induced cell migration, proliferation and collagen synthesis. Mechanically, inhibition of PKM2 ameliorated cardiac remodelling by suppressing TGF‐β/Smad2/3 and Jak2/Stat3 signalling pathway through oxidative stress. Therefore, PKM2 plays an adverse role in cardiac remodelling diseases (Figure 7B).

Cardiac fibrosis is pivotal in cardiac remodelling and heart failure. Persistent cardiac fibrosis causes an increase in cardiac muscle stiffness, eventually leads to cardiac dysfunction.^23^ PKM2 is a key enzyme involved in the aerobic glycolysis of rapidly proliferation cells, which involves in fibroblast function and fibrosis.^24^, ^25^, ^26^ Increasing evidence suggests that PKM2 contributes to pathological fibrosis process in different diseases. In chronic kidney diseases, overexpression of PKM2 remarkably induces myofibroblast activation and renal interstitial fibrosis, and inhibition of PKM2 by shikonin reverses these detrimental effects.^27^ PKM2 is elevated in diabetic model mouse lungs and hearts, which regulates glucose‐induced lung fibrogenesis.^28^ The expression of PKM2 is markedly increased in TAC‐induced hypertrophic hearts.^29^ In our study, we also found that PKM2 expression was elevated in Ang‐II‐infused remodelling mouse hearts and cardiac fibroblasts. Inhibition of PKM2 by shikonin ameliorated cardiac fibrosis, hypertrophy and dysfunction in Ang‐II‐induced mouse cardiac remodelling. In vitro, suppression of PKM2 alleviated Ang‐II‐induced migration, proliferation and collagen synthesis. Hence, PKM2 acts as a pro‐fibrotic role in cardiac remodelling.

TGF‐β/Smad2/3 and Jak2/Stat3 signalling are primary pathways in fibrogenesis and majorly contribute to reactive fibrosis in Ang‐II‐induced cardiac remodelling.^30^ TGF‐β, one of the best characterized fibrogenic growth factors, is markedly and consistently activated by Ang II in fibrotic animal hearts.^31^ Overexpression of TGF‐β stimulates the deposition of excessive collagen in cardiac interstitium and eventually induces ventricular fibrosis.^32^ In addition, blockade of TGF‐β prevents cardiac fibrosis in pressure overload model.^33^ Smad2/3, reported as TGF‐β transcriptional activators, promotes the activation of cardiac fibroblasts into myofibroblasts and aggravate pathological cardiac fibrosis.^34^ We found that suppression of PKM2 restrained the activation of TGF‐β/Smad2/3 signalling pathway. Numerous data indicate that the Jak2/Stat3 signalling pathway is involved in the development of cardiac fibrosis.^35^ Selective inhibition of Stat3 phosphorylation ameliorates left‐atrial fibrosis in mouse MI model through inhibiting collagen synthesis.^6^ Our results showed that suppression of PKM2 inhibited phosphorylation of Jak2 and Stat3. These data indicate that PKM2‐activated aggravates cardiac fibrosis by targeting TGF‐β/Smad2/3 and Jak2/Stat3 signalling pathways.

Oxidative stress plays an important role in the pathophysiology of myocardial fibrosis.^36^ In an in vivo, ROS promotes collagen synthesis and deposition in fructose‐ and high glucose‐induced myocardial fibrosis.^37^, ^38^ In adult rat cardiac fibroblasts, the generation of ROS induced by Ang II also stimulates collagen production.^39^ MDA, namely lipid peroxidation, is also a widely accepted biomarker of oxidative stress.^40^ In our study, inhibition of PKM2 reduced Ang‐II‐induced ROS production and MDA levels. Diverse‐specific and ‐nonspecific anti‐oxidant defence systems exist to scavenge ROS. GSH and GPx4, major anti‐oxidants, protect cells against oxidative stress by decreasing the generation of ROS.^41^, ^42^ We found that Ang II decreased GSH/GSSG ratio and GPx4 levels, while inhibition of PKM2 opposed these detrimental effects. Thus, we suggest that PKM2 exacerbates cardiac fibrosis through oxidative stress.

Oxidative stress was reported to activate TGFβ1‐Smad2/3 and Jak2/Stat3 signalling. Our study here revealed an elevation of ROS generation and TGFβ1‐Smad2/3 and Jak2/Stat3signalling in response to Ang II treatment. We also observed that inhibition of oxidative stress by NAC suppressed Ang‐II‐induced TGFβ/Smad2/3 and Jak2/Stat3 activation in NRCFs. Combined with previous findings, we can speculate that shikonin attenuates Ang‐II‐induced cardiac fibrosis via TGF‐β/Smad2/3 and Jak2/Stat3 through oxidative stress. Interestingly, TGFβ1‐Smad2/3 and Jak2/Stat3 signalling activation were also reported to directly promote oxidative stress,^43^, ^44^, ^45^ thus suggesting a vicious cycle between TGFβ1‐Smad2/3 and Jak2/Stat3 signalling activation and ROS overproduction in Ang‐II‐induced pro‐fibrotic response.

In summary, we have discovered that PKM2 promotes angiotensin‐II‐induced cardiac remodelling by activating TGF‐β/Smad2/3 and Jak2/Stat3 pathways through oxidative stress. We propose negative modulation of PKM2 as a potential therapeutic strategy for the treatment of cardiac remodelling diseases.

## CONFLICT OF INTEREST

None declared.

## AUTHOR CONTRIBUTIONS

**Xiyu Zhang:** Conceptualization (equal); Data curation (equal); Formal analysis (equal); Writing‐original draft (lead). **Cuiting Zheng:** Conceptualization (equal); Data curation (equal); Formal analysis (equal). **Zhenqiang Gao:** Investigation (equal). **Lingling Wang:** Investigation (equal). **Chen Chen:** Investigation (equal). **Yuanyuan Zheng:** Investigation (equal). **Yan Meng:** Funding acquisition (lead); Writing‐review & editing (lead).

## Supporting information

Fig S1

Fig S2

## Data Availability

The data that support the findings of this study are available on request from the corresponding author.

## References

[1] Weber KT , Sun Y , Gerling IC , Guntaka RV . Regression of established cardiac fibrosis in hypertensive heart disease. Am J Hypertens. 2017;30(11):1049‐1052. 28379281 10.1093/ajh/hpx054

[2] Gjesdal O , Bluemke DA , Lima JA . Cardiac remodeling at the population level–risk factors, screening, and outcomes. Nat Rev Cardiol. 2011;8(12):673‐685. 22027657 10.1038/nrcardio.2011.154

[3] Schiattarella GG , Hill JA . Inhibition of hypertrophy is a good therapeutic strategy in ventricular pressure overload. Circulation. 2015;131(16):1435‐1447. 25901069 10.1161/CIRCULATIONAHA.115.013894 PMC4408778

[4] Su SA , Yang D , Wu Y , et al. EphrinB2 regulates cardiac fibrosis through modulating the interaction of Stat3 and TGF‐beta/Smad3 signaling. Circ Res. 2017;121(6):617‐627. 28743805 10.1161/CIRCRESAHA.117.311045

[5] Sakata Y , Chancey AL , Divakaran VG , Sekiguchi K , Sivasubramanian N , Mann DL . Transforming growth factor‐beta receptor antagonism attenuates myocardial fibrosis in mice with cardiac‐restricted overexpression of tumor necrosis factor. Basic Res Cardiol. 2008;103(1):60‐68. 18034274 10.1007/s00395-007-0689-5 PMC3872069

[6] Chen Y , Surinkaew S , Naud P , et al. JAK‐STAT signalling and the atrial fibrillation promoting fibrotic substrate. Cardiovasc Res. 2017;113(3):310‐320. 28158495 10.1093/cvr/cvx004 PMC5852635

[7] Pizzino G , Irrera N , Cucinotta M , et al. Oxidative stress: harms and benefits for human health. Oxid Med Cell Longev. 2017;2017:8416763. 28819546 10.1155/2017/8416763 PMC5551541

[8] Duni A , Liakopoulos V , Roumeliotis S , Peschos D , Dounousi E . Oxidative stress in the pathogenesis and evolution of chronic kidney disease: untangling ariadne's thread. Int J Mol Sci. 2019;20(15):3711. 10.3390/ijms20153711 PMC6695865 31362427

[9] Sinha N , Dabla PK . Oxidative stress and antioxidants in hypertension‐a current review. Curr Hypertens Rev. 2015;11(2):132‐142. 26022210 10.2174/1573402111666150529130922

[10] Klaunig JE . Oxidative stress and cancer. Curr Pharm Des. 2018;24(40):4771‐4778. 30767733 10.2174/1381612825666190215121712

[11] Dai DF , Johnson SC , Villarin JJ , et al. Mitochondrial oxidative stress mediates angiotensin II‐induced cardiac hypertrophy and Galphaq overexpression‐induced heart failure. Circ Res. 2011;108(7):837‐846. 21311045 10.1161/CIRCRESAHA.110.232306 PMC3785241

[12] Dixon SJ . Ferroptosis: bug or feature? Immunol Rev. 2017;277(1):150‐157. 28462529 10.1111/imr.12533

[13] Li J , Li S , Guo J , et al. Natural product Micheliolide (MCL) irreversibly activates pyruvate kinase M2 and suppresses leukemia. J Med Chem. 2018;61(9):4155‐4164. 29641204 10.1021/acs.jmedchem.8b00241 PMC5949721

[14] Palsson‐McDermott EM , Curtis AM , Goel G , et al. Pyruvate kinase M2 regulates Hif‐1alpha activity and IL‐1beta induction and is a critical determinant of the warburg effect in LPS‐activated macrophages. Cell Metab. 2015;21(1):65‐80. 25565206 10.1016/j.cmet.2014.12.005 PMC5198835

[15] Dayton TL , Jacks T , Vander Heiden MG . PKM2, cancer metabolism, and the road ahead. EMBO Rep. 2016;17(12):1721‐1730. 27856534 10.15252/embr.201643300 PMC5283597

[16] Liang J , Cao R , Wang X , et al. Mitochondrial PKM2 regulates oxidative stress‐induced apoptosis by stabilizing Bcl2. Cell Res. 2017;27(3):329‐351. 28035139 10.1038/cr.2016.159 PMC5339831

[17] Bi HL , Zhang XL , Zhang YL , et al. The deubiquitinase UCHL1 regulates cardiac hypertrophy by stabilizing epidermal growth factor receptor. Sci Adv. 2020;6(16):eaax4826. 32494592 10.1126/sciadv.aax4826 PMC7164950

[18] Sun Q , Chen X , Ma J , et al. Mammalian target of rapamycin up‐regulation of pyruvate kinase isoenzyme type M2 is critical for aerobic glycolysis and tumor growth. Proc Natl Acad Sci U S A. 2011;108(10):4129‐4134. 21325052 10.1073/pnas.1014769108 PMC3054028

[19] Latella G . Redox imbalance in intestinal fibrosis: beware of the TGFbeta‐1, ROS, and Nrf2 connection. Dig Dis Sci. 2018;63(2):312‐320. 29273848 10.1007/s10620-017-4887-1

[20] Chen RR , Fan XH , Chen G , et al. Irisin attenuates angiotensin II‐induced cardiac fibrosis via Nrf2 mediated inhibition of ROS/ TGFbeta1/Smad2/3 signaling axis. Chem Biol Interact. 2019;302:11‐21. 30703374 10.1016/j.cbi.2019.01.031

[21] Simon AR , Rai U , Fanburg BL , Cochran BH . Activation of the JAK‐STAT pathway by reactive oxygen species. Am J Physiol. 1998;275(6):C1640‐C1652. 9843726 10.1152/ajpcell.1998.275.6.C1640

[22] Severgnini M , Takahashi S , Rozo LM , et al. Activation of the STAT pathway in acute lung injury. Am J Physiol Lung Cell Mol Physiol. 2004;286(6):L1282‐L1292. 14729509 10.1152/ajplung.00349.2003

[23] Delaunay M , Osman H , Kaiser S , Diviani D . The role of cyclic AMP signaling in cardiac fibrosis. Cells. 2019;9(1):69. 10.3390/cells9010069 PMC7016856 31888098

[24] Iansante V , Choy PM , Fung SW , et al. PARP14 promotes the Warburg effect in hepatocellular carcinoma by inhibiting JNK1‐dependent PKM2 phosphorylation and activation. Nat Commun. 2015;6:7882. 26258887 10.1038/ncomms8882 PMC4918319

[25] Murphy MK , Motz KM , Ding D , et al. Targeting metabolic abnormalities to reverse fibrosis in iatrogenic laryngotracheal stenosis. Laryngoscope. 2018;128(2):E59‐E67. 28940431 10.1002/lary.26893 PMC5771827

[26] Ryu C , Sun H , Gulati M , et al. Extracellular mitochondrial DNA is generated by fibroblasts and predicts death in idiopathic pulmonary fibrosis. Am J Respir Crit Care Med. 2017;196(12):1571‐1581. 28783377 10.1164/rccm.201612-2480OC PMC5754440

[27] Li Q , Qin Z , Nie F , et al. Metabolic reprogramming in keloid fibroblasts: aerobic glycolysis and a novel therapeutic strategy. Biochem Biophys Res Commun. 2018;496(2):641‐647. 29337061 10.1016/j.bbrc.2018.01.068

[28] Bhedi CD , Nasirova S , Toksoz D , et al. Glycolysis regulated transglutaminase 2 activation in cardiopulmonary fibrogenic remodeling. FASEB J. 2020;34(1):930‐944. 31914588 10.1096/fj.201902155R PMC6956703

[29] Liu R , Kenney JW , Manousopoulou A , et al. Quantitative Non‐canonical Amino Acid Tagging (QuaNCAT) proteomics identifies distinct patterns of protein synthesis rapidly induced by hypertrophic agents in cardiomyocytes, revealing new aspects of metabolic remodeling. Mol Cell Proteomics. 2016;15(10):3170‐3189. 27512079 10.1074/mcp.M115.054312 PMC5054342

[30] Eid RA , Alkhateeb MA , El‐Kott AF , et al. A high‐fat diet rich in corn oil induces cardiac fibrosis in rats by activating JAK2/STAT3 and subsequent activation of ANG II/TGF‐1beta/Smad3 pathway: the role of ROS and IL‐6 trans‐signaling. J Food Biochem. 2019;43(8):e12952. 31368573 10.1111/jfbc.12952

[31] Cao F , Li Z , Ding WM , Yan L , Zhao QY . LncRNA PVT1 regulates atrial fibrosis via miR‐128‐3p‐SP1‐TGF‐beta1‐Smad axis in atrial fibrillation. Mol Med. 2019;25(1):7. 30894138 10.1186/s10020-019-0074-5 PMC6425687

[32] Rosenkranz S , Flesch M , Amann K , et al. Alterations of beta‐adrenergic signaling and cardiac hypertrophy in transgenic mice overexpressing TGF‐beta(1). Am J Physiol Heart Circ Physiol. 2002;283(3):H1253‐H1262. 12181157 10.1152/ajpheart.00578.2001

[33] Kuwahara F , Kai H , Tokuda K , et al. Transforming growth factor‐beta function blocking prevents myocardial fibrosis and diastolic dysfunction in pressure‐overloaded rats. Circulation. 2002;106(1):130‐135. 12093782 10.1161/01.cir.0000020689.12472.e0

[34] Khalil H , Kanisicak O , Prasad V , et al. Fibroblast‐specific TGF‐beta‐Smad2/3 signaling underlies cardiac fibrosis. J Clin Invest. 2017;127(10):3770‐3783. 28891814 10.1172/JCI94753 PMC5617658

[35] Zhang Y , Zhang L , Fan X , et al. Captopril attenuates TAC‐induced heart failure via inhibiting Wnt3a/beta‐catenin and Jak2/Stat3 pathways. Biomed Pharmacother. 2019;113:108780. 30889487 10.1016/j.biopha.2019.108780

[36] Wang LP , Fan SJ , Li SM , Wang XJ , Gao JL , Yang XH . Oxidative stress promotes myocardial fibrosis by upregulating KCa3.1 channel expression in AGT‐REN double transgenic hypertensive mice. Pflugers Arch. 2017;469(9):1061‐1071. 28455747 10.1007/s00424-017-1984-0

[37] Zhang Y , Zhang Y . Toll‐like receptor‐6 (TLR6) deficient mice are protected from myocardial fibrosis induced by high fructose feeding through anti‐oxidant and inflammatory signaling pathway. Biochem Biophys Res Commun. 2016;473(2):388‐395. 26940740 10.1016/j.bbrc.2016.02.111

[38] Evangelista I , Nuti R , Picchioni T , Dotta F , Palazzuoli A . Molecular dysfunction and phenotypic derangement in diabetic cardiomyopathy. Int J Mol Sci. 2019;20(13):3264. 10.3390/ijms20133264 PMC6651260 31269778

[39] Lijnen P , Papparella I , Petrov V , Semplicini A , Fagard R . Angiotensin II‐stimulated collagen production in cardiac fibroblasts is mediated by reactive oxygen species. J Hypertens. 2006;24(4):757‐766. 16531806 10.1097/01.hjh.0000217860.04994.54

[40] Turut H , Ciralik H , Kilinc M , Ozbag D , Imrek SS . Effects of early administration of dexamethasone, N‐acetylcysteine and aprotinin on inflammatory and oxidant‐antioxidant status after lung contusion in rats. Injury. 2009;40(5):521‐527. 18707685 10.1016/j.injury.2008.05.001

[41] Dai J , Weinberg RS , Waxman S , Jing Y . Malignant cells can be sensitized to undergo growth inhibition and apoptosis by arsenic trioxide through modulation of the glutathione redox system. Blood. 1999;93(1):268‐277. 9864170

[42] Feng H , Stockwell BR . Unsolved mysteries: how does lipid peroxidation cause ferroptosis? PLoS Biol. 2018;16(5):e2006203. 29795546 10.1371/journal.pbio.2006203 PMC5991413

[43] Wang Y , Yu X , Song H , et al. The STAT‐ROS cycle extends IFNinduced cancer cell apoptosis. Int J Oncol. 2018;52(1):305‐313. 29115415 10.3892/ijo.2017.4196

[44] Black D , Lyman S , Qian T , et al. Transforming growth factor beta mediates hepatocyte apoptosis through Smad3 generation of reactive oxygen species. Biochimie. 2007;89(12):1464‐1473. 17936489 10.1016/j.biochi.2007.09.001 PMC2151473

[45] Krylatov AV , Maslov LN , Voronkov NS , et al. Reactive oxygen species as intracellular signaling molecules in the cardiovascular system. Curr Cardiol Rev. 2018;14(4):290‐300. 29962348 10.2174/1573403X14666180702152436 PMC6300799

