# Supplementary figures and images for: PKM2 promotes angiotensin‐II‐induced cardiac remodelling by activating TGF‐β/Smad2/3 and Jak2/Stat3 pathways through oxidative stress
Source: J Cell Mol Med. 2021 Oct 23;25(22):10711–23. doi: 10.1111/jcmm.17007 (PMC8581335; doi:10.1111/jcmm.17007)

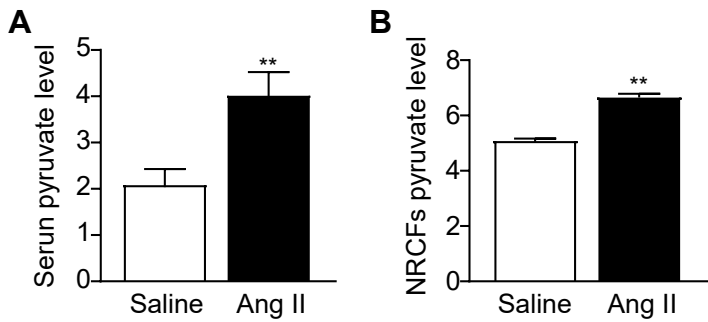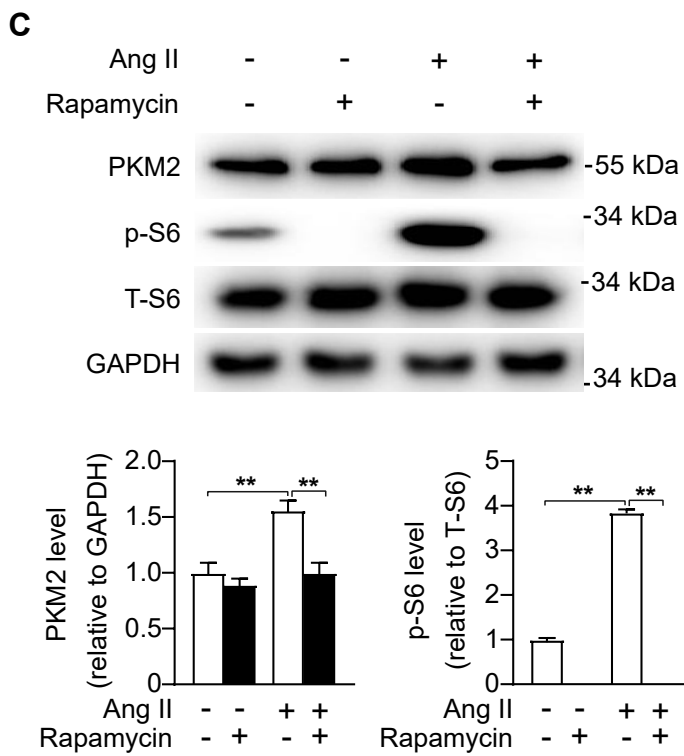

Supplement: Supplementary file 1 — Fig S1 [file JCMM-25-10711-s002.pdf]

**A**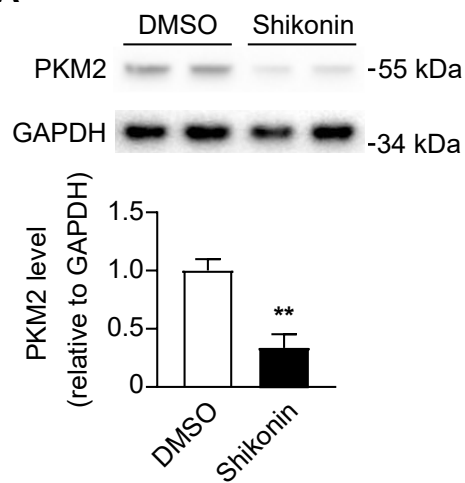**B**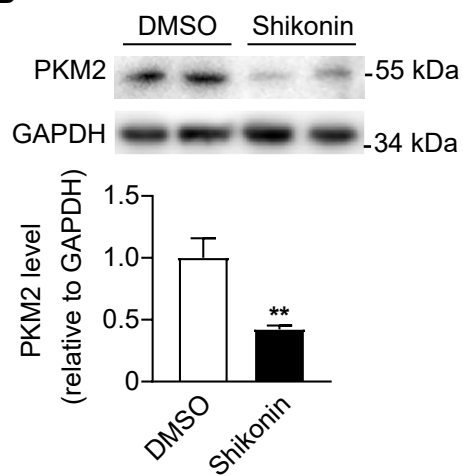

Supplement: Supplementary file 2 — Fig S2 [file JCMM-25-10711-s001.pdf]
